# Quality indicators for ambulatory colectomy: literature search and expert consensus

**DOI:** 10.1007/s00464-023-10660-3

**Published:** 2024-02-05

**Authors:** Ellen Coeckelberghs, Gabriele Bislenghi, Albert Wolthuis, An Teunkens, Geertrui Dewinter, Steve Coppens, Kris Vanhaecht, André D’Hoore

**Affiliations:** 1 Leuven Institute for Healthcare Policy, Department of Public Health and Primary Care, KU Leuven – University of Leuven, Leuven, Belgium; 2 Department of Abdominal Surgery, University Hospitals Leuven, Leuven, Belgium; 3 Department of Anaesthesiology, University Hospitals Leuven, Leuven, Belgium; 4 Department of Quality Management, University Hospitals Leuven, Leuven, Belgium

**Keywords:** Colectomy, Ambulatory care, Quality indicators, Abdominal surgery, Quality of care

## Abstract

**Background:**

Care for patients undergoing elective colectomy has become increasingly standardized using Enhanced Recovery Programs (ERP). ERP, encorporating minimally invasive surgery (MIS), decreased postoperative morbidity and length of stay (LOS). However, disruptive changes are needed to safely introduce colectomy in an ambulatory or same-day discharge (SDD) setting. Few research groups showed the feasibility of ambulatory colectomy. So far, no minimum standards for the quality of care of this procedure have been defined. This study aims to identify quality indicators (QIs) that assess the quality of care for ambulatory colectomy.

**Methods:**

A literature search was performed to identify recommendations for ambulatory colectomy. Based on that search, a set of QIs was identified and categorized into seven domains: preparation of the patient (pre-admission), anesthesia, surgery, in-hospital monitoring, home monitoring, feasibility, and clinical outcomes. This list was presented to a panel of international experts (surgeons and anesthesiologists) in a 1 round Delphi to assess the relevance of the proposed indicators.

**Results:**

Based on the literature search (2010–2021), 3841 results were screened on title and abstract for relevant information. Nine papers were withheld to identify the first set of QIs (*n* = 155). After excluding duplicates and outdated QIs, this longlist was narrowed down to 88 indicators. Afterward, consensus was reached in a 1 round Delphi on a final list of 32 QIs, aiming to be a comprehensive set to evaluate the quality of ambulatory colectomy care.

**Conclusion:**

We propose a list of 32 QI to guide and evaluate the implementation of ambulatory colectomy.

In recent decades, care for patients undergoing elective colectomy has become increasingly standardized using Enhanced Recovery Programs (ERP).[1, 2] Due to minimally invasive surgery (MIS) techniques and ERP, postoperative morbidity and length of stay (LOS) have steadily decreased [3–6]. A further decrease in LOS will require a disruptive change to allow a same-day discharge (SDD) or one-night stay. Ambulatory or SDD surgery is defined as a patient being discharged home on the same calendar date as the surgery date. Early discharge and recovery in the comforts of one’s home yields many advantages: improved quality of sleep, emotional support from family and friends, earlier return to individual food and beverage preferences, avoidance of exposure to hospital-associated infections [7].

In an ambulatory setting, both surgery and anesthesiology play a pivotal role in the management of the patient. There is a need for standardized postoperative multimodal pain management protocols. Early discharge is often compromised by ad hoc administration of opioids [7]. Therefore, adequate pain management with only oral analgesics is considered one of the most important discharge criteria after colorectal surgery [8]. From a surgical point of view, the focus lies on low impact surgery, including minimally invasive surgery, but also low-pressure laparoscopy. A third condition is adequate post-discharge remote follow-up [9]. To avoid diagnostic and therapeutic delay, potential complications, both minor and major adverse events, should be captured rapidly at home in the early postoperative phase [7].

Different factors drive the recent increase in ambulatory surgery. During the COVID-19 pandemic, the push towards ambulatory procedures was driven by the patients’ requests for early discharge and the limited hospital bed capacity. Moreover, remote control monitoring has gained increasing interest and implementation in the past few years. The pandemic accelerated the transition to monitoring and therapy based on patient risks and needs[10], which should be further implemented in elective surgery. In the following years, we expect continuing growth in ambulatory colorectal surgery.

To organize the most optimal care for patients, there is a need to coordinate and evaluate the full care pathway of the patient undergoing ambulatory colectomy. A care pathway is defined as a complex intervention for the mutual decision-making and organisation of care pathways for a well-defined group of patients during a well-defined period [11]. In an ambulatory setting, the focus lies on transmural pathways where the follow-up process is shifted to primary care providers or facilities. Moreover, the quality of care and possible barriers or bottlenecks should be identified and evaluated throughout the full transmural care pathway. This study aims to develop indicators enabling monitoring and evaluating the quality of ambulatory colorectal resections from admission until follow-up in the home setting.

## Methods

**Fig. 1:**
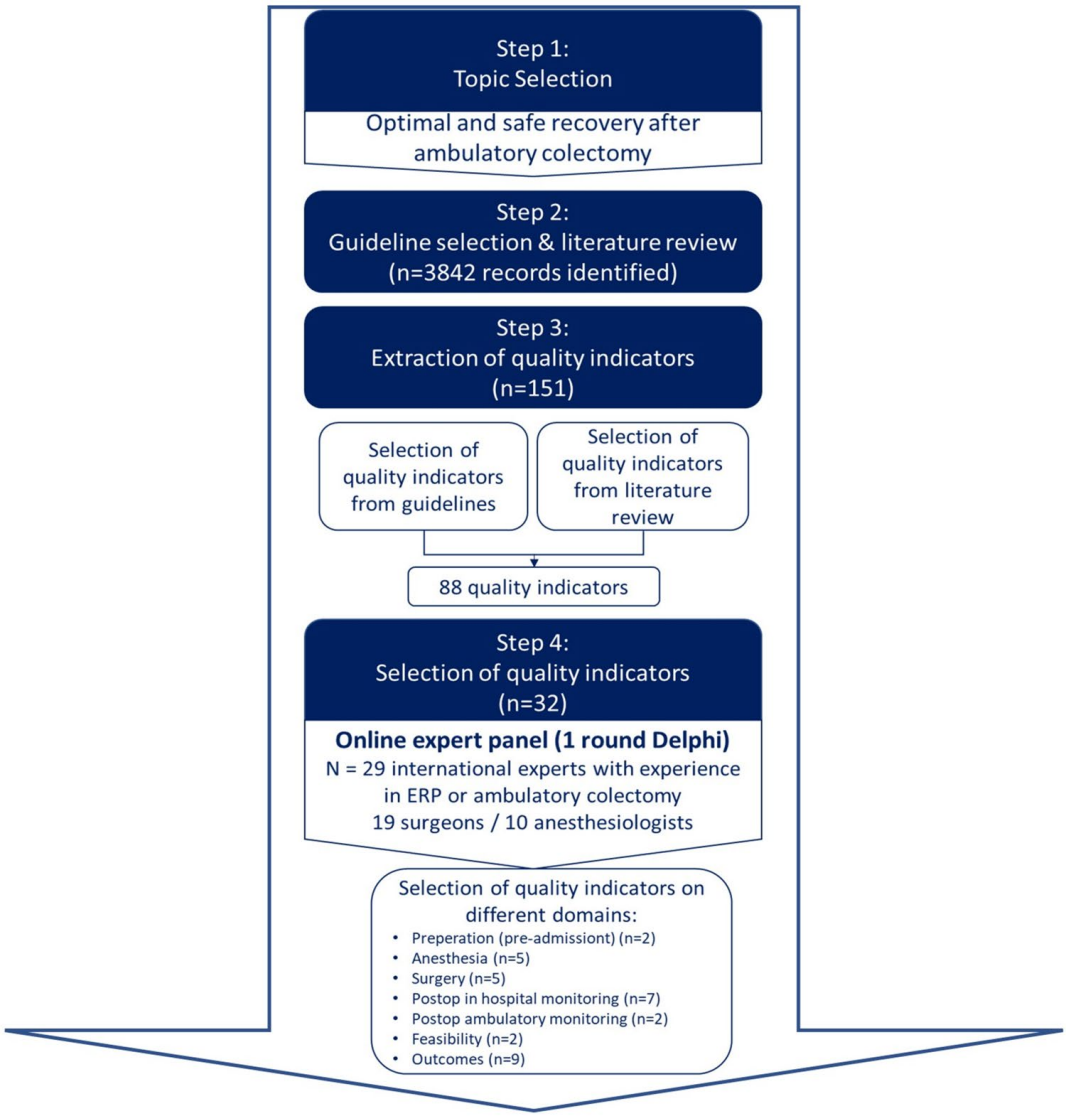
Study flowchart: Step 1 to 4 of the stepwise approach for a guideline-based development of quality indicators (adapted from Kotter et al.)

A systematic and transparent approach is necessary to develop key indicators assessing the quality of care, i.e. quality indicators (QIs) [12]. For this reason, QIs will be used that are scientifically acceptable, feasible, clinically relevant, and usable[13] and additionally enable discrimination [14]. Therefore, the stepwise approach for guideline-based development of QI proposed by Kotter et al. was used [15]. In the first step, topics were selected and identified. As a second step, quality indicators from peer-reviewed literature were selected. Third, quality indicators from the guidelines and literature were extracted. In the fourth step, QIs are selected by an expert panel by performing a one-round Delphi. Experts were asked to indicate, on a 10-point rating scale, the relevance of each indicator for evaluating the quality of ambulatory colorectal resections. Consensus was defined as agreement if at least 75% of the respondents rated an indicator as relevant (score 8,9, or 10). The Delphi round responses were presented using the mean score and differences between surgeons and anesthesiologists were analyzed using Student *t* tests for continuous data. In the original model of Kotter, the fifth step consists of a practice test that has to be conducted, followed by the implementation of the QI. However, the current study will be limited to the selection of QI, i.e., steps 1–4 according to this model. For this study, no IRB approval or written consent was needed (Fig. 1).

## Results

### Step 1: topic selection

Topic selection, “optimal and safe recovery after ambulatory colectomy,” was performed by a researcher and a small group of colorectal surgeons and anesthesiologists involved in ambulatory care (EC, GB, AW, AT, GD, ADH). A rigorous patient selection preoperatively is of prime importance for patients undergoing ambulatory colectomy. This selection should be based on the patient’s history, comorbidities, and type of surgery. Eligibility criteria are age between 18 and 75 years; ASA classification ≤ 2; BMI < 30; use of home monitoring app; distance to the hospital: ≤ 50 km; colectomy for diverticulitis or colonic cancer; no mental illness and good social support. Patients with significant comorbidities (including insulin-dependent diabetes, chronic renal failure, cardiac and respiratory comorbidities that require in-hospital monitoring); creation of a stoma; taking anticoagulants, antidepressants, opiates (regular use), and consuming > 20 alcoholic consumptions/week were excluded. Economic impact and impact on quality of life were considered.

### Step 2: literature review and selection of guidelines

**Fig. 2:**
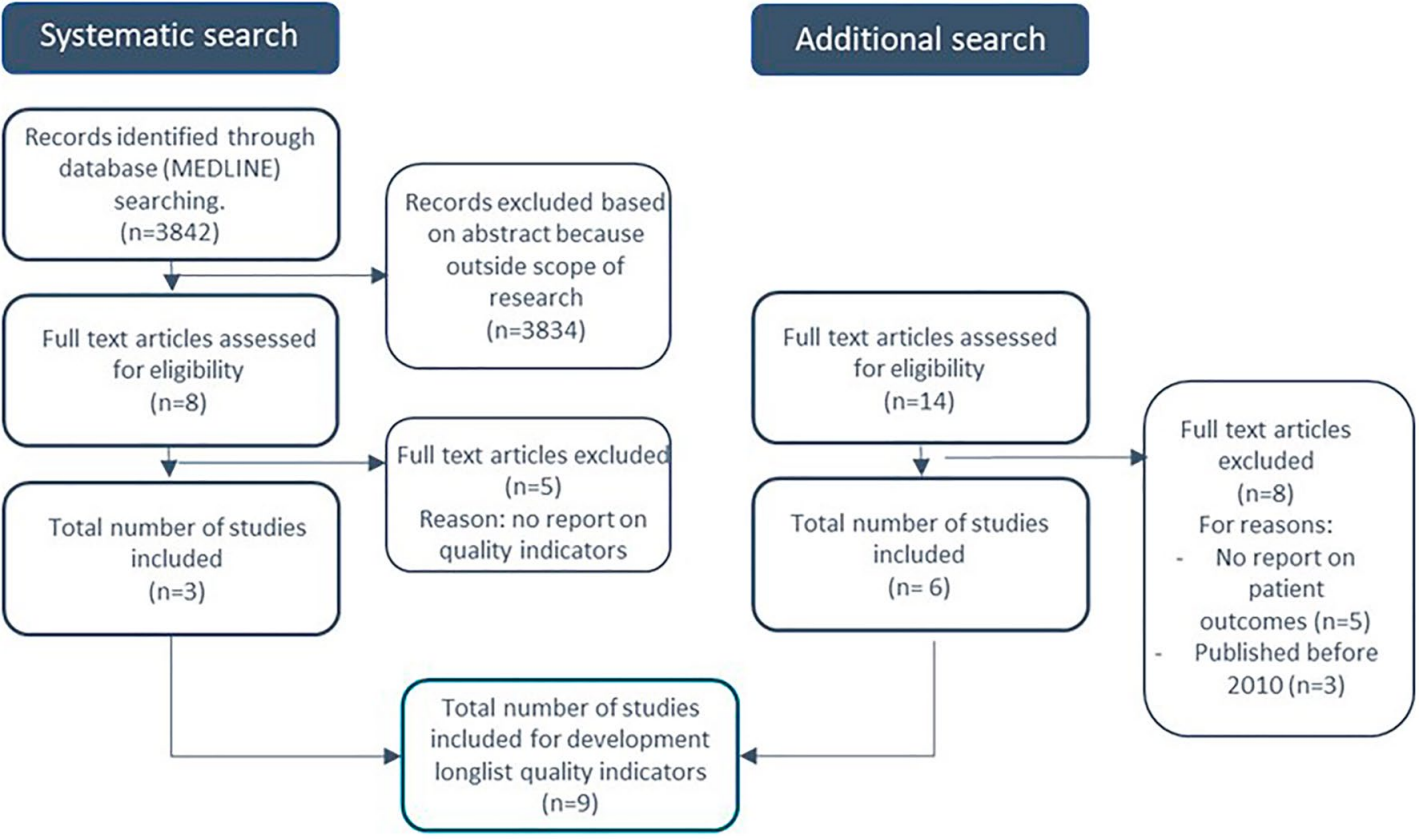
Flowchart of the article selection process

The principal researchers, EC and GB, conducted an extensive literature review to identify all available evidence for integrating the evidence-based ambulatory colectomy care pathway. A Medline search was conducted by exploring the following search terms: (“Quality Indicators, Health Care” [Mesh] OR “patient-reported outcome measures” [Mesh] OR “Outcome and Process Assessment, Health Care” [Mesh] OR “Quality Assurance, Health Care” [Mesh]) AND (“Colectomy” [Mesh] OR colectom*) between January 1, 2010, and March 31, 2021. Only articles including human subjects and English-written articles were included. Three thousand eight hundred forty-one results were screened on title and abstract for relevant information (Fig. 2).

### Step 3: extraction of quality indicators

Based on the literature search, a first set of QIs (*n* = 151) was identified and categorized into seven domains that cover the whole peri-operative care pathway of the patient: preparation of the patient (pre-admission), anesthesia, surgery, in-hospital monitoring, home monitoring, feasibility, and clinical outcomes. After excluding duplicates, this longlist was narrowed down to 65 indicators. Internal experts reviewed this list and added another 23 QIs that are relevant and specific to ambulatory abdominal care.

### Step 4: final selection of the indicators

**Fig. 3:**
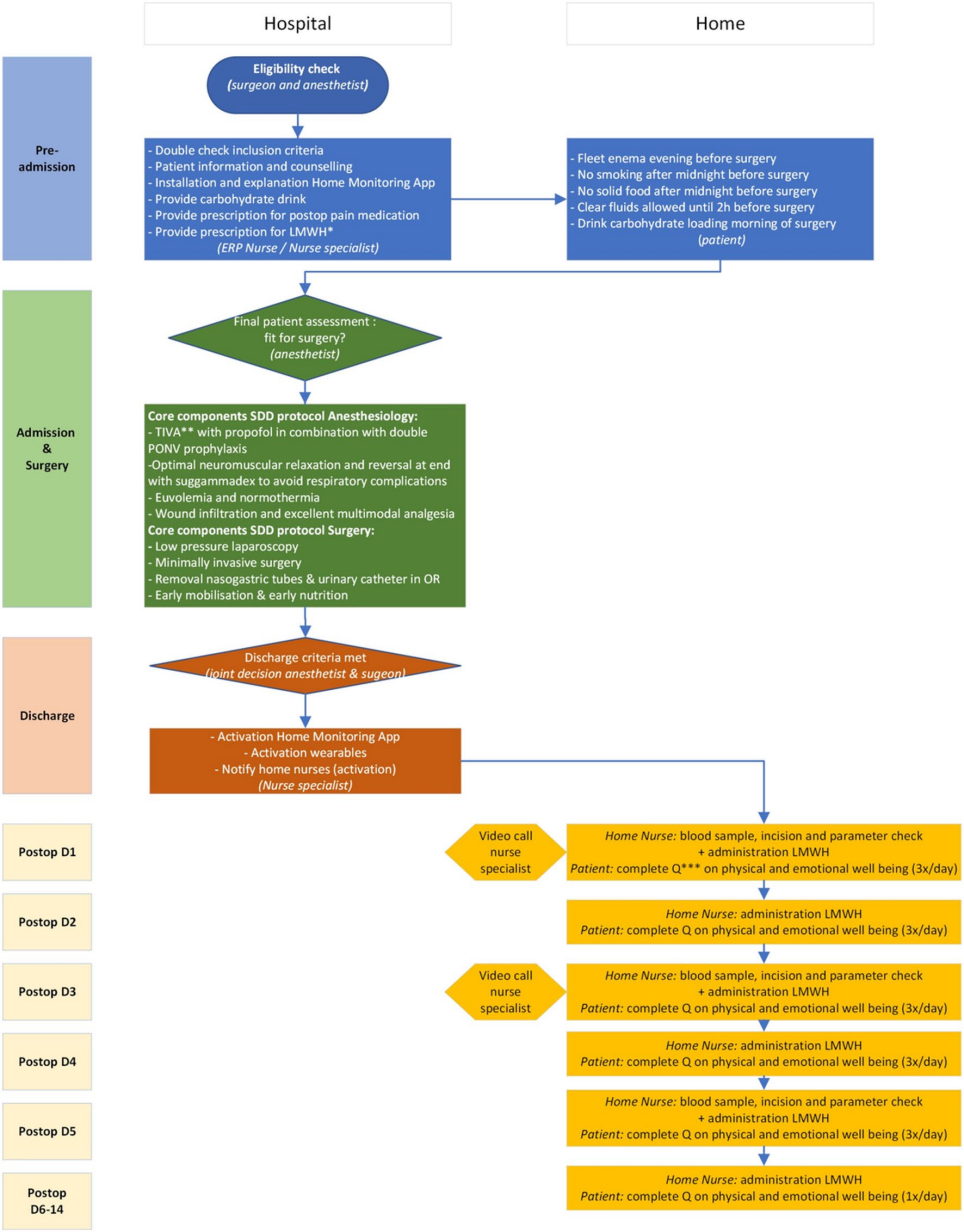
Flowchart of the ambulatory colectomy care process. *Low molecular weight heparin; **Total intravenous anesthesia; ***Questionnaire

In a fourth step, a panel of 29 international experts [Belgium (*n* = 19); Denmark (*n* = 1); France (*n* = 1); Germany (*n* = 2); Italy (*n* = 1); Spain (*n* = 1); Switzerland (*n* = 2); UK (*n* = 1); USA (*n* = 1)] was asked to score these 88 QIs on their clinical relevance. This panel consisted of 19 surgeons and ten anesthesiologists with experience with rapid recovery programs or ambulatory colectomy. Consensus was reached for 27 of 88 indicators (30.6%). The highest consensus was reached for indicators for readmission rate (100%), 30-day mortality (96.7%), pre-admission patient education, administration of standard PONV protocol, number of intraoperative adverse events, anastomotic leakage, and postoperative bleeding (93.3%). There was no significant difference in scoring the relevance between the surgeons and anesthesiologists, except for three indicators: low-pressure laparoscopy, duration of the procedure, and surgical site infection. Anesthesiologists scored the relevance of low-pressure laparoscopy and duration of the procedure higher than surgeons, whereas surgeons found the prevalence of surgical site infections more important. Next, a small group of experts evaluated the relevance and inclusion of the extracted QIs. Consequently, a consensus was reached on a final list of 32 QIs, as listed in Table 1. For the use of the indicator set in daily clinical practice, a specific flowchart was designed (Fig. 3).

**Table 1:** Quality indicators for optimal and safe recovery after ambulatory colectomy

Indicator	Mean score surgeon (*n* = 19)	Mean score Anesthesiologist (*n* = 10)	*p*-value
*Preparation of the patient (pre-admission)*			
Pre-admission information, education, and counseling: dedicated preop counseling	9.4	9.1	0.169
Preoperative anesthesiology consult (including comorbidity assessment)	8.4	9.7	0.962
*Anesthesia (intraoperative)*			
Standard protocol for PONV	9.0	8.8	0.625
Standard care for normothermia	8.7	8.7	0.905
Body temperature below 36 °C at the end of surgery	8.3	8.9	0.186
DVT prophylaxis	7.6	8.7	0.556
Intraoperative metamizole & paracetamol & 1 dose of opioids	6.5	6.9	0.559
*Surgery (intraoperative)*			
Minimally invasive surgery	9.2	9.2	0.219
Low-pressure laparoscopy (between 6 and 10 mmHg)	6.1	8.9	**0.002**
Duration of the procedure	7.3	9.2	**0.018**
Intraoperative surgical AE (e.g., major bleeding, small bowel injury, soiling, ischemia)	9.2	9.0	0.605
Conversion rate	9.1	9.1	0.787
*Process: postoperative in-hospital monitoring*			
Pain evaluation (VAS Score): every 2 h	7.3	8.4	0.068
Spontaneously urinating before discharge	8.8	8.0	0.272
Opioid avoidance	8.0	8.0	1.000
Drinking before discharge	8.2	8.8	0.856
Walking ± 5 m before discharge (at least transfer bed toilet)	8.8	8.6	0.592
Review of significant adverse events in the hospital	8.3	8.6	0.919
Unscheduled in-hospital consult	8.4	8.4	0.879
*Process: postoperative ambulatory monitoring*			
Blood sample (CRP, hemoglobin, and renal function) on postop days 1, 3 and 5	5.9	7.4	0.269
Review of significant adverse events at home (Dindo-Clavien ≥ 2)	8.5	8.4	0.555
*Feasibility*			
Unplanned admission to the hospital (outside one night stay)	8.6	8.4	0.348
Unplanned return to the operating room	9.3	8.8	0.157
*Outcomes*			
Anastomotic leakage requiring intervention	9.2	9.0	0.166
Surgical site infection	9.2	8.5	**0.025**
Postoperative bleeding	8.2	8.8	0.621
Readmission rate	8.6	8.9	0.757
Complication rate (Dindo-Clavien/Cumulative Complication Index)	9.4	9.1	0.574
Acute urinary retention	8.8	9.0	0.703
In-hospital mortality	7.9	8.1	0.844
30-day mortality	9.6	9.1	0.147
90-day mortality	9.0	9.0	0.837

Bold values indicate significance at *p* < 0.05

## Discussion

In 2015, Gignoux and colleagues were the first to demonstrate the feasibility of ambulatory colectomy [16]. In 2018, the same research group published the first series of 157 consecutive patients undergoing ambulatory colectomy. They showed feasibility, safety, and reproducibility in a selected group of patients [17]. Since different other series have been published [18, 19]. In our center, the transition from ERP (multi-day hospital stay) to SDD has been initiated in November 2022. However, up to date, there is no comprehensive set of QIs to monitor and evaluate the quality of ambulatory colectomy in terms of safety and feasibility. For this reason, guidelines are required for the further implementation of ambulatory colectomy and a comprehensive list of QI is timely. Only QIs published in peer-reviewed journals were withheld and classified into categories using the patient care pathway: preparation of the patient (pre-admission), intraoperative anesthesiologic indicators, intraoperative surgical indicators, postoperative in-hospital monitoring, postoperative ambulatory monitoring, feasibility, and outcomes. A one-round Delphi was organized to integrate expert opinion effectively. Our study results in a core set of 32 QIs based on evidence and expert opinion. Twenty-nine (91%) of the QIs were not rated significantly different between surgeons and anesthesiologists, indicating that both professional groups support the set. The importance of this is emphasized in the literature. In an ambulatory setting, next to patient and family/support education and adequate postoperative remote follow-up, surgery and anesthesiology play a pivotal role in the management of the patient and the success of the transmural care pathway [7]. Adequate patient education and follow-up must be performed using home monitoring devices and applications [7, 9, 16]. Anesthesiology should focus on improvements in multimodal analgesia [20, 21], nausea prevention [19], glucose and temperature control [19], amongst others. Conversely, surgery should focus on minimally invasive techniques [22] and short operating time [7] with minimal risk for intraoperative adverse events.

According to Vu et al., the key components of a routine SDD are patient education, a multidisciplinary pathway, and advanced surgical techniques [22]. These components are covered in the QI set described in the present study.

Possible barriers to implementing SDD are described in the literature as inexperience with remote control monitoring in the early postoperative recovery period, the need for multidisciplinary support, insufficient support at home, and the impact of distance from the hospital [7]. Lee et al. state that possible resistance from surgeons and clinicians and their concerns about patient expectations are potential barriers [20]. Moreover, the safety issue of ambulatory colectomy is of utmost importance. Postoperative adverse events like anastomotic leakage, postoperative bleeding, urine retention, inadequate oral pain control, and postoperative ileus are most commonly reported [18, 19, 22]. Therefore, they must be captured as early as possible to avoid postoperative morbidity and mortality. These adverse events are all part of the QIs in our set.

### Strengths and limitations

Previously conducted studies, however, show that despite consensus on content, the adherence to guidelines is low [6], and change is hard, even in ERP [23, 24]. This underscores the importance of evaluating the care pathway of colectomy when transitioning or ‘changing’ from traditional ERP to SDD. As SDD colectomy is an emerging specific care process, specific literature is rather limited. Therefore, the set of QIs developed in this study is a first attempt to provide clinicians and other healthcare professionals with new benchmark criteria. The level of evidence to define the QIs in the actual study is very high, and due to the robust methodology used, our study might serve as a hinge to improve the quality of care. However, with ongoing experience and published outcome data this QI set will be adapted and become more specific related to SDD. The current set is in line with QIs for traditional ERP since the same principles are used: preoperative patient optimization, minimally invasive surgical techniques and multimodal pain control being the key elements. Additionally, more specific to SDD is the concept of remote patient monitoring en entails relying on adapted technology like capturing vital parameters using wearables and an interactive personalized application to monitor physical en emotional well-being.

The validation was performed by a panel of 29 experts out of 8 countries and thus provides an internationally validated set of quality indicators for future studies. Moreover, these quality indicators can be used for benchmarking initiatives on a national and international level, finally leading to quality improvement in ambulatory care.

Another strength of our study is that the whole care pathway of the patient is covered, from preoperative information and counseling to postoperative home monitoring and outcomes. Finally, some QIs that are not based on evidence but are evaluated as high quality by international experts are included in the set, like, for instance, low-pressure laparoscopy. As an international group of experts validated the QIs by consensus, they are not dependent on area or region.

One of the limitations of our study is that we only performed a one-round Delphi to reach a consensus. Moreover, among the experts, we only included surgeons and anesthesiologists. Nevertheless, nurses, paramedics, general practitioners, primary care nurses and patients are essential in this care pathways; our Delphi did not include them. However, this does not imply we underestimate their role in the success of ambulatory colectomy. Finally, in our study, the fifth (practice test) and sixth step (implementation) of the original model of Kotter have not been performed yet. A final limitation is that we focus only on the patient outcomes that are related to safety and effectiveness. In our set of QIs, indicators that are linked to the wider social construct around the people involved in receiving and providing care (KIN) are not included. For example, the impact on the team (in-hospital and transmural), but also the caregivers was not addressed. In future research, we should take into account the multidimensional quality model [25], which embraces a more holistic view on quality of healthcare.

## Conclusion

Based on a literature review and Delphi, a set of 32 QI to monitor the safe and effective implementation of daycare colectomy is provided. Further research should focus on the impact on the patient, the (transmural) team providing care and patients’ relatives. Secure remote patient monitoring will play a pivotal role in transmural care pathways, not being limited to colectomy.
